# Supplementary material for: Inhibiting perovskite decomposition by a creeper-inspired strategy enables efficient and stable perovskite solar cells
Source: Nat Commun. 2024 Jun 18;15:5223. doi: 10.1038/s41467-024-49617-y (PMC11189488; doi:10.1038/s41467-024-49617-y)
Supplement: Supplementary file 1 — Supplementary Information [file 41467_2024_49617_MOESM1_ESM.pdf]

Supplementary information

Inhibiting perovskite decomposition by a creeper-inspired strategy enables efficient and stable perovskite solar cells

Shuxian Du[1,2], Hao Huang[1,2], Zhineng Lan[1], Peng Cui[1], Liang Li[1], Min Wang[1], Shujie Qu[1], Luyao Yan[1], Changxu Sun[1], Yingying Yang[1], Xinxin Wang[1]&Meicheng Li[1]*

[1]State Key Laboratory of Alternate Electrical Power System with Renewable Energy Sources, School of New Energy, North China Electric Power University, Beijing 102206, China.

[2]These authors contributed equally: Shuxian Du, Hao Huang.

[*]Corresponding author: Meicheng Li (mcli@ncepu.edu.cn)

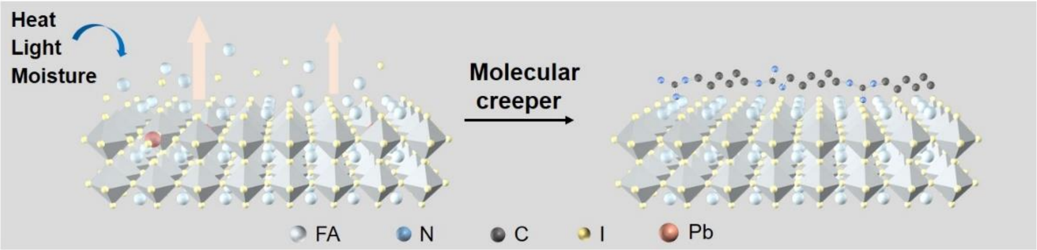

Supplementary Fig. 1 Schematic illustration of the stabilization of perovskite by molecular creeper under the conditions of heat, light, and moisture.

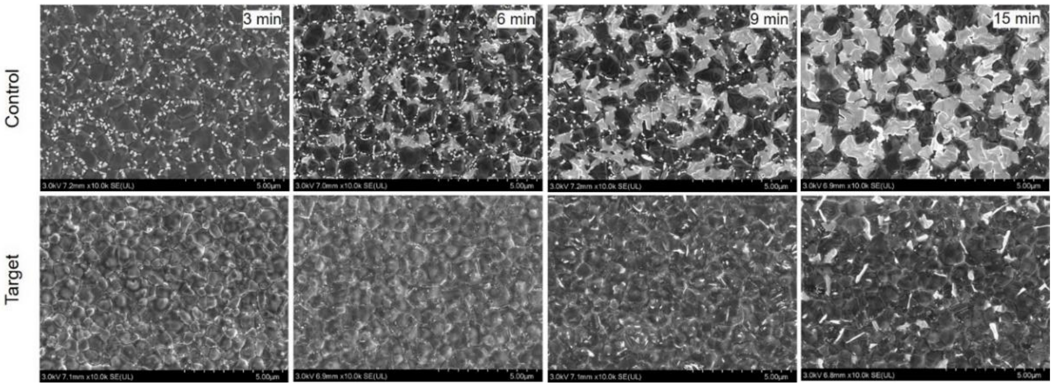

Supplementary Fig. 2 The surface SEM images of control and target perovskite films.

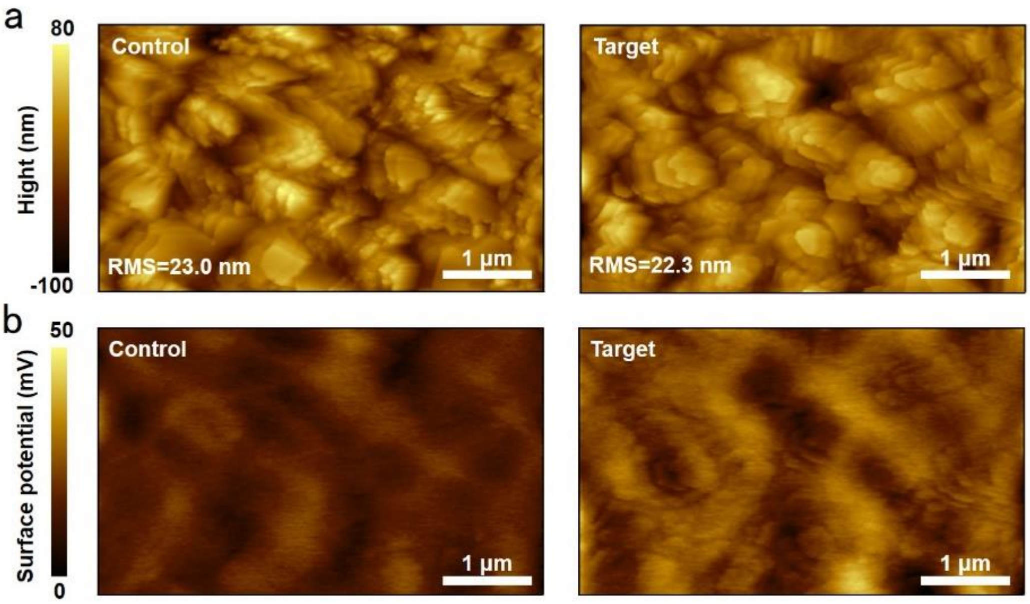

Supplementary Fig. 3 (a) The AFM images of control and target perovskite films. (b) The KPFM images of control and target perovskite films.

Supplementary Note 1. As shown in Supplementary Fig. 3a, compared to the target film, although the AFM image is difficult to show the residual PbI_2 on the control film, we can still notice that there are many tiny particles located at the grain boundary on the control film, which may correspond to the PbI_2. From the image of surface potential shown in Supplementary Fig. 3b, we can find that the control film possesses a lower surface potential, which should result from the residual PbI_2. The results of KPFM can validate that the perovskite undergoes decomposition accompanied by the generation of PbI_2 in the annealing process, and the creeper-inspired strategy can effectively inhibit this decomposition.

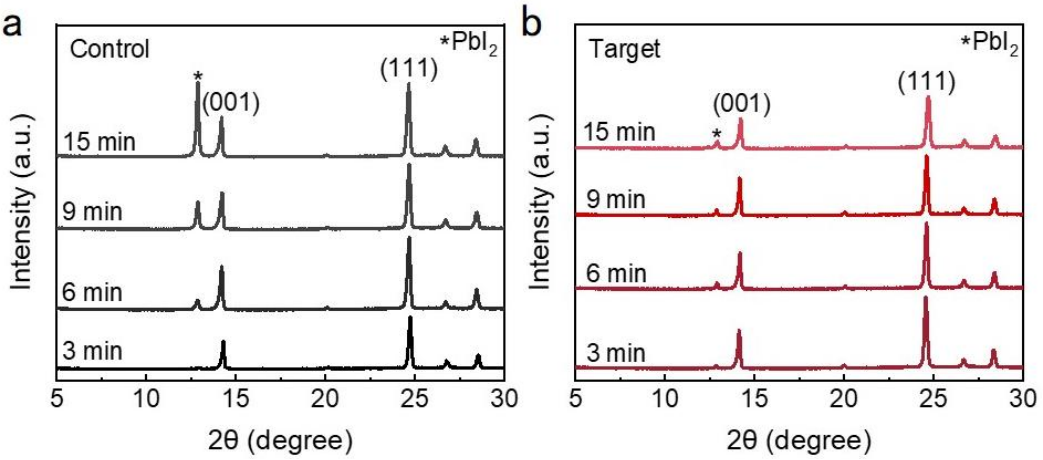

Supplementary Fig. 4 XRD of (a) control and (b) target perovskite films with different annealing times.

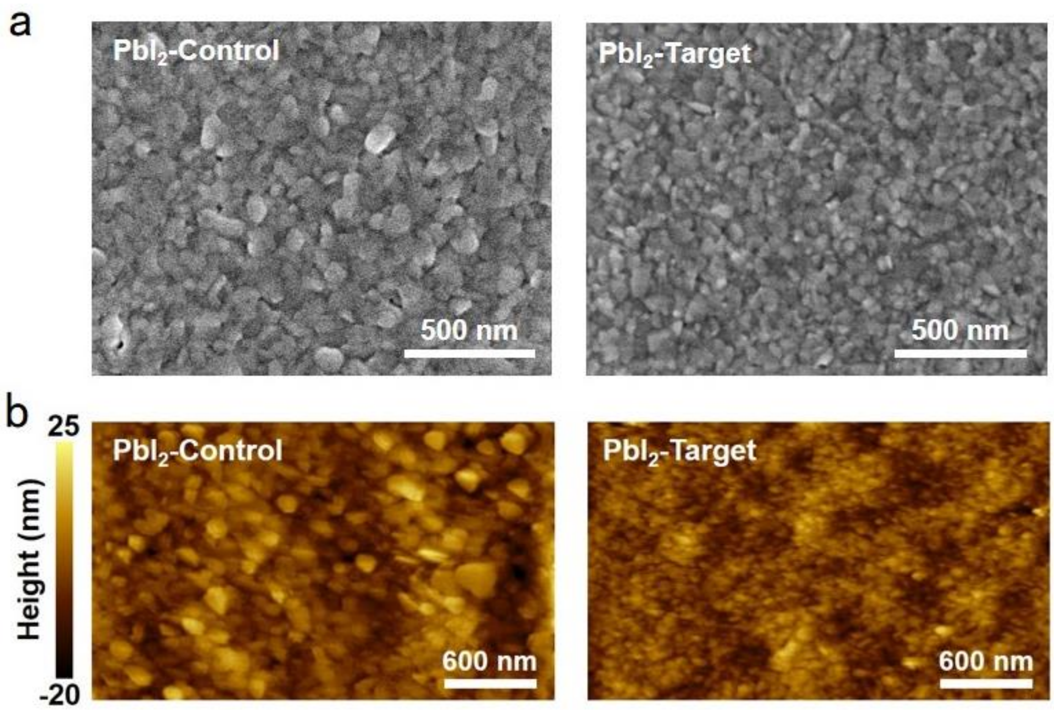

Supplementary Fig. 5 (a) Surface SEM images and (b) AFM images of control and target PbI_2 films.

Supplementary Note 2. After incorporating PHMG into PbI_2 precursor, it can be seen the PbI_2 film has a smaller grain through measurements of SEM and AFM. The smaller grains are conducive to the full reaction between PbI_2 with organic salt.

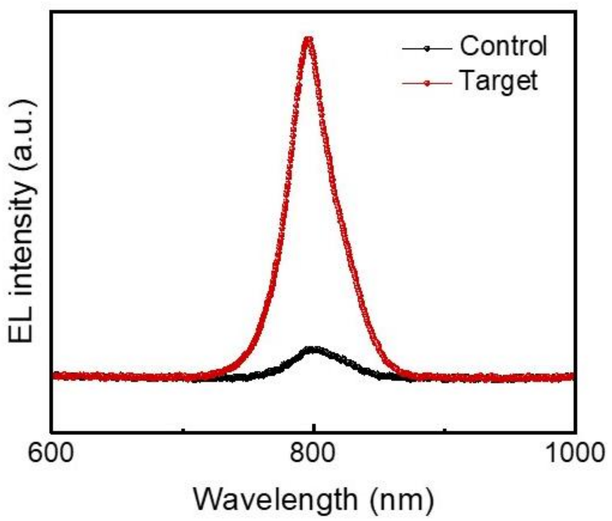

Supplementary Fig. 6 Electroluminescence (EL) measurement of control and target PSCs.

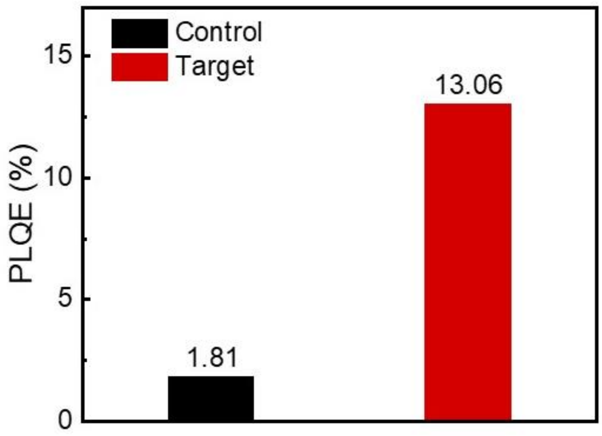

Supplementary Fig. 7 Photoluminescence quantum efficiency (PLQE) of control and target PSCs. The excitation wavelength is 470 nm.

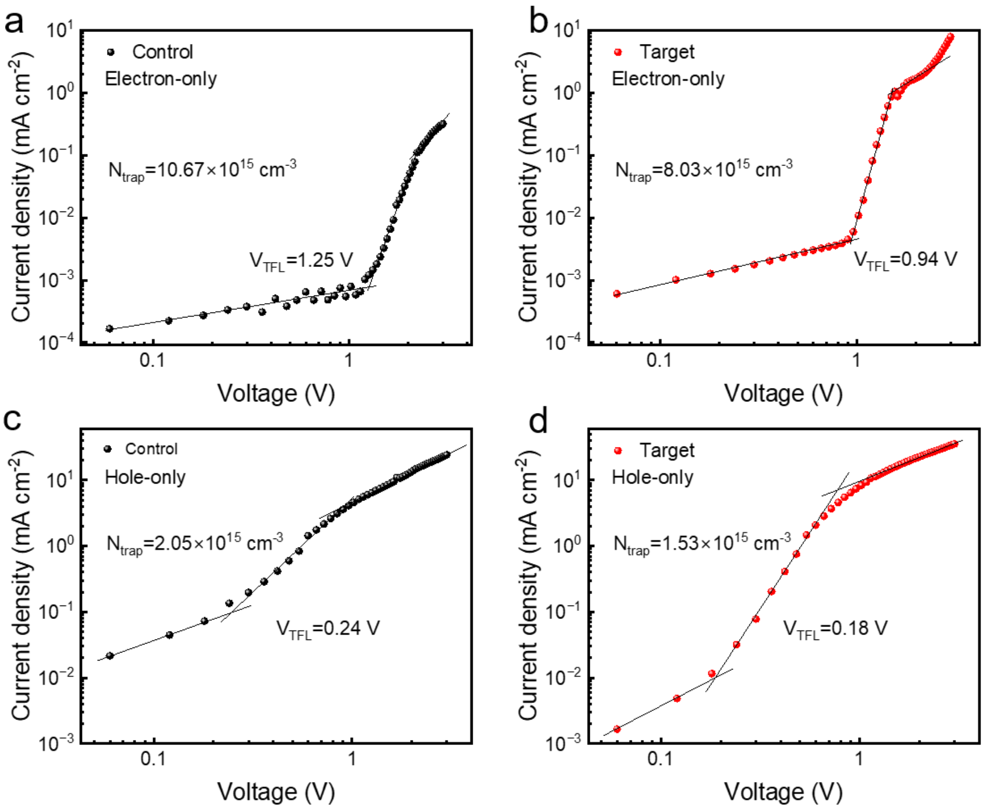

Supplementary Fig. 8 The dark current-voltage characteristics of control and target devices. (a, b) electron-only devices with the structure FTO/TiO_2/perovskite (passivation layer)/PCBM/Au, (c, d) hole-only devices with the structure FTO/PEDOT: PSS /perovskite (passivation layer)/Spiro-OMeTAD/Au. V_{TFL} represents the trap-filled limit voltage.

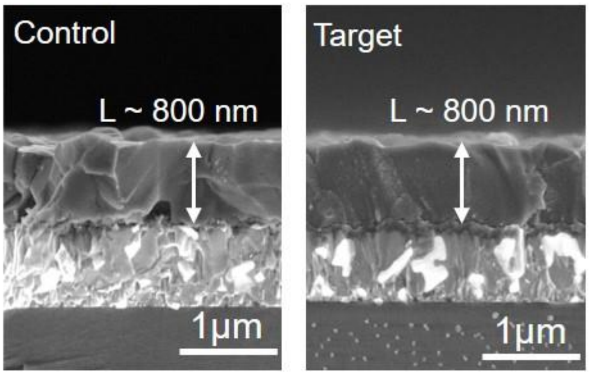

Supplementary Fig. 9 Cross-sectional SEM images of control and target perovskite films fabricated by two-step spin-coating. The thickness (L) of the perovskite films is ~800 nm.

Supplementary Note 3. Calculation of defect density.

The trap density (N_{trap}) was calculated according to the following equation:

$$N_{trap} = \frac{2\varepsilon\varepsilon_0 V_{TFL}}{eL^2}$$

where e is the electron charge, L is the thickness of perovskite film, ε is the relative dielectric constant, and ε_0 is the vacuum permittivity and V_{TFL} is the trap-filled limit voltage. In our PSCs, the V_{TFL} of both perovskite films is 1.25 V and 0.94 V in the electron-only device, while 0.24 V and 0.18 V in the hole-only device.

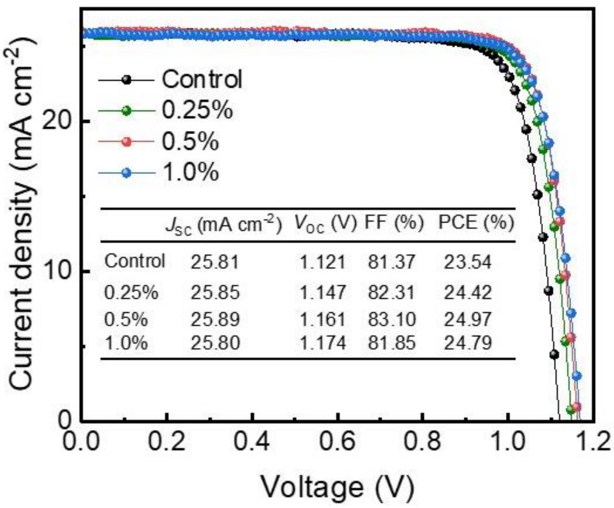

Supplementary Fig. 10 Optimum *J*-*V* curves of the PSCs with different PHMG modification concentrations in the reverse scan direction.

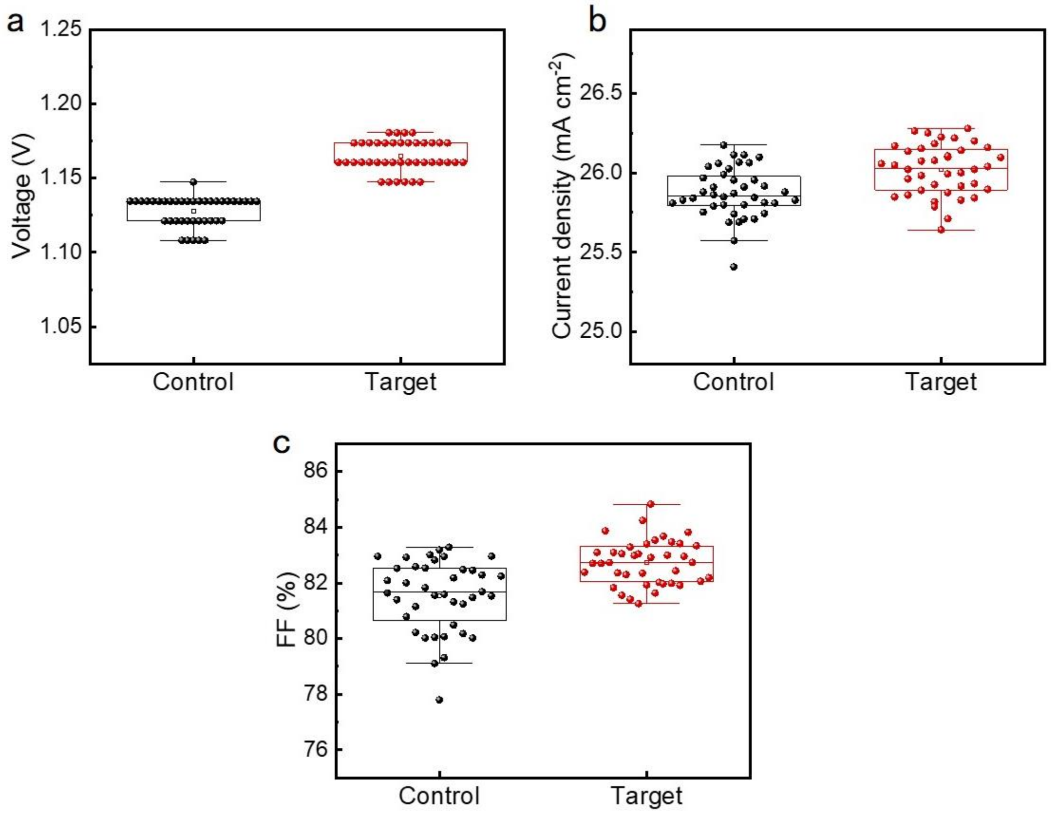

Supplementary Fig. 11 (a) V_{OC}, (b) J_{SC} and (c) FF of control and target PSCs fabricated by two-step method.

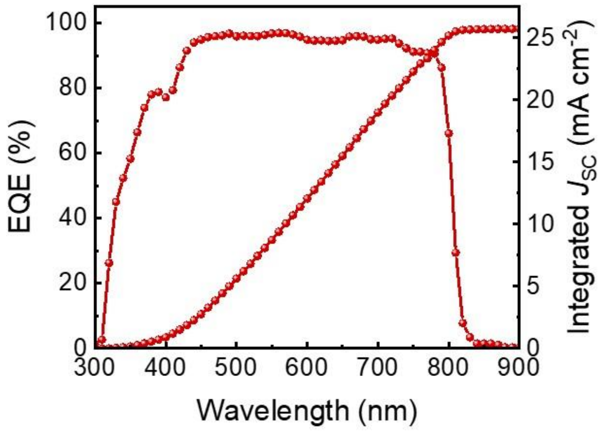

Supplementary Fig. 12 The external quantum efficiency (EQE) of target PSCs**.** The integrated J_{SC} is 25.72 mA cm^{-2}.

Appendix: Summary of the Report
Report No.: GXgf2024-00285
Client: North China Electric Power University
Sample: Perovskite Solar Cell
Type/Model: Perovskite Solar Cell
DUT S/N: 6-M2
Manufacturer:North China Electric Power University
Sample temperature: (25±1)℃
Date of Test: 01/25/2024
Environmental conditions: (20±1) ℃, RH (12±2) %
Mask: An aperture area of 8.053 mm² (M2#, Certificate No.: CDjc2022-00151)

The test has been conducted by the PV Metrology Lab of NIM (National Institute of Metrology, China). Measurement of irradiance intensity and all other measurements are traceable to the International System of Units (SI). Data in this report apply only at the time of the test for the sample. For more details, please refer to the text of the report.

	Area (mm²)	I_{sc}(mA)	V_{oc}(V)	P_{max}(mW)
Reverse Scan	8.053	2.104	1.181	2.042
Forward Scan	8.053	2.102	1.183	2.036
	I_{max}(mA)	V_{max}(V)	FF (%)	η (%)
Reverse Scan	2.002	1.020	82.18	25.36
Forward Scan	1.996	1.020	81.89	25.28

I-V Characterization Methods:
JJF 1622-2017: Calibration Specification of Solar Cells: Photoelectric Properties
Reference Solar Cell:
Type: Mono-Si
Solar Simulator:
Classification: A+AA+ (Double-light source: Xeon and Halogen);
Total irradiance: 1000 W/m² based on I_{sc} of the above Reference Solar Cell.

Supplementary Fig. 13 Certified results by the National Institute of Metrology, China (NIM, China). This certified report and corresponding permission are obtained from the NIM, China.

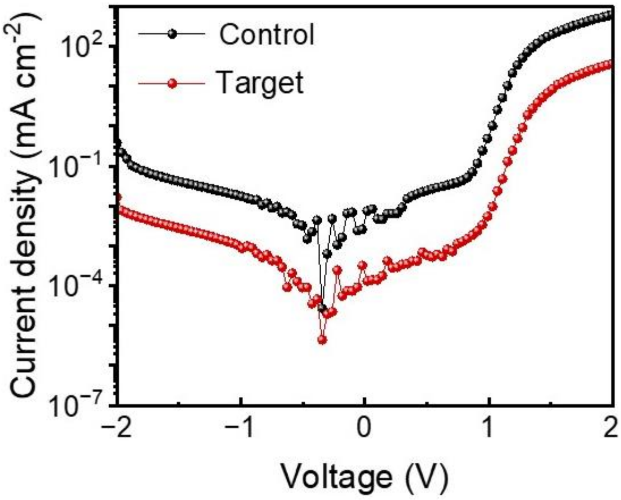

Supplementary Fig. 14 Dark *J-V* curves of control and target PSCs. The voltage scan range is from -2 to 2 V.

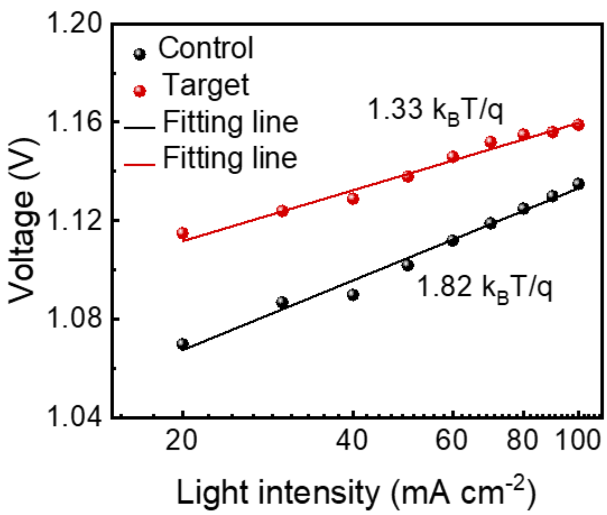

Supplementary Fig. 15 The dependence of V_{OC} on the light intensity of control and target PSCs. The ideality factor (n_{ID}) was calculated according to the equation $n_{ID} = \frac{q}{k_B T}\frac{d\,V_{OC}}{d\ln(I)}$. In this equation, k_B is the Boltzmann constant, T is the thermodynamic temperature, and q is the electron charge. After calculation, the n_{ID} are 1.82 and 1.33 $k_B T/q$ for control and target perovskite PSCs.

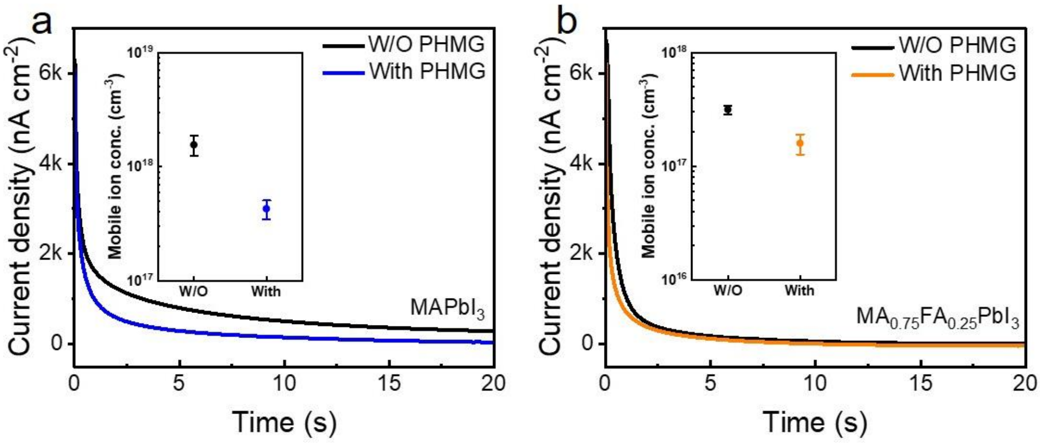

Supplementary Fig. 16 Transient ion-migration currents and mobile ion concentrations of fresh (a) $MAPbI_3$ (b) $MA_{0.75}FA_{0.25}PbI_3$ devices. 1.1 V forward bias applied to the devices is removed. Five devices were tested in each group.

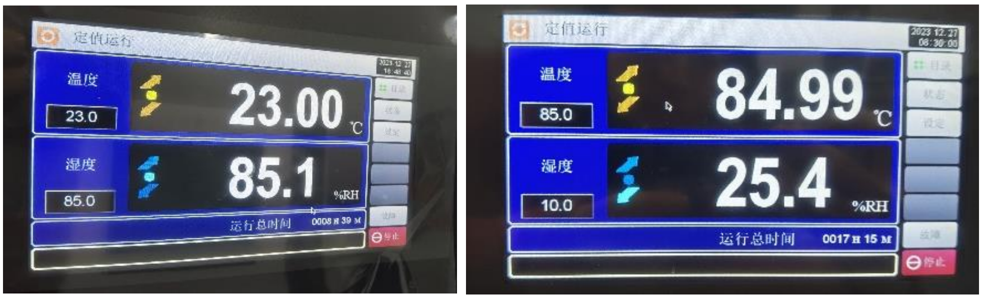

Supplementary Fig. 17 Accelerated aging conditions (~85% RH with ~23℃ and ~85℃ with ~25% RH)

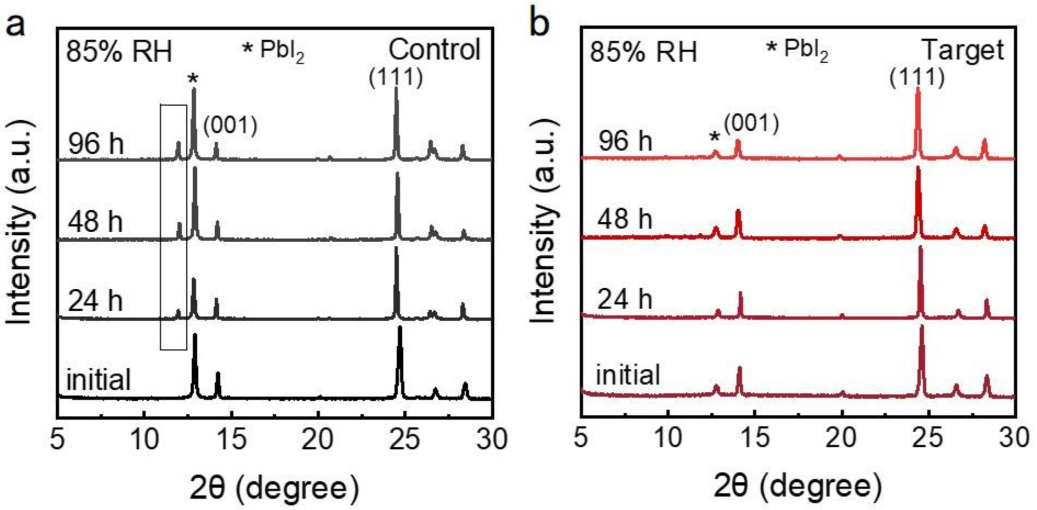

Supplementary Fig. 18 The XRD evolution of (a) control and (b) target perovskite films under accelerating aging at 85% RH with room temperature.

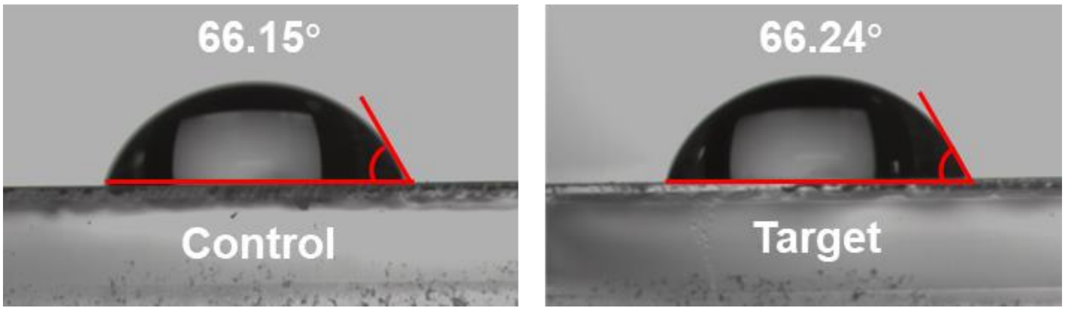

Supplementary Fig. 19 The H_2O contact angle of control and target perovskite films.

Supplementary Table 1. A List of reported representative PCE of planar PSCs with TiO_2 as ETL.

Structure	Method of TiO_2 film	PCE (%) In house	Year	PCE (%) Certified	Reference
FTO/TiO_2/ $Cs_{0.05}FA_{0.85}MA_{0.10}Pb(I_{0.97}Br_{0.03})_3$	Chemical bath deposition	24.13	2022	/	1
FTO/TiO_2&PCBA/$FA_{0.97}Cs_{0.03}PbI_{2.91}Br_{0.09}$	Spin-coating	24.25	2022	23.7	2
FTO/LD-TiO_2/$FA_{0.92}MA_{0.08}PbI_3$	Chemical bath deposition	24.81	2022	24.5	3
FTO/TiO_2/$FA_xMA_{1-x}PbI_3$	Chemical bath deposition	24.33	2023	/	4
FTO/TiO_2/$FAPbI_3$	Chemical bath deposition	24.67	2023	/	5
FTO/TiO_2/$FAPbI_3$	Chemical bath deposition	25.30	2023	24.8	6
FTO/TiO_2/F/$Cs_xFA_yMA_{1-x-y}Pb(I_zBr_{1-z})_3$	Chemical bath deposition	25.30	2023	/	7
FTO/CdTe-TiO_2/ $Cs_{0.05}FA_{0.81}MA_{0.14}PbI_{2.57}Br_{0.43}$	Chemical bath deposition	25.02	2023	/	8
FTO/TiO_2/$(FAPbI_3)_{0.95}(MAPbBr_3)_{0.05}$	Chemical bath deposition	24.45	2023	/	9
FTO/TiO_2/AS/$FAPbI_3$	Chemical bath deposition	24.78	2023	/	10
FTO/TiO_2/$FA_xMA_{1-x}PbI_3$	Chemical bath deposition	25.0	2023	/	11
FTO/HFSTA-TiO_2/$FAPbI_3$	Chemical bath deposition	25.03	2023	/	12
FTO/TiO_2-CSBA/$FAPbI_3$	Chemical bath deposition	25.32	2024	25.32	13
FTO/TiO_2/$FAPbI_3$	Chemical bath deposition	25.42	2024	25.36	This work

Supplementary Table 2. The mobile ion concentration of control and target PSCs.

Sample	Mobile ion concentration ($\times 10^{16}$ cm^{-3})					Average
Control	15.70	13.80	23.00	14.20	19.70	17.28
Target	6.68	5.56	5.20	6.54	6.36	6.07

Supplementary Note 4. The transient ion-migration currents measurement was performed by an electrochemical workstation under pure dark condition. A bias of 1.1 V was applied for 20 s and then removed. The mobile ion concentration of control and target PSCs was calculated according to the equation $n = \frac{Integrated\ J_{SC}}{el}$. In this equation, e is the electron charge and l is the thickness of perovskite films.

Supplementary Table 3. The mobile ion concentration of $MAPbI_3$-PSCs without and with PHMG.

Sample	Mobile ion concentration ($\times 10^{17}$ cm^{-3})					Average
W/O PHMG	15.80	17.40	19.30	11.70	13.10	15.46
With PHMG	3.83	3.81	4.09	3.85	5.65	4.25

Supplementary Table 4. The mobile ion concentration of $MA_{0.75}FA_{0.25}PbI_3$-PSCs without and with PHMG.

Sample	Mobile ion concentration ($\times 10^{17}$ cm^{-3})					Average
W/O PHMG	2.83	3.29	2.97	3.03	3.54	3.13
With PHMG	1.12	1.57	1.48	1.90	1.85	1.58

Supplementary Table 5. The initial values of PCE in stability tests.

Sample	Long-term stability	Operational stability	85°C heating in ambient air	85% RH at room temperature
Control	23.83%	19.26%	19.46%	19.92%
Target	24.41%	20.48%	20.73%	20.50%

Supplementary References

1. Du X*, et al.* Synergistic crystallization and passivation by a single molecular additive for high-performance perovskite solar cells. *Adv Mater.* **34**, e2204098 (2022).
2. Li Y*, et al.* Efficient, stable formamidinium-cesium perovskite solar cells and minimodules enabled by crystallization regulation. *Joule* **6**, 676-689 (2022).
3. Huang H*, et al.* 24.8%-efficient planar perovskite solar cells via ligand-engineered TiO_2 deposition. *Joule* **6**, 2186-2202 (2022).
4. Liu L*, et al.* Manipulating electron density distribution of nicotinamide derivatives toward defect passivation in perovskite solar cells. *Adv. Energy Mater.* **13**, 2300610 (2023).
5. Su H*, et al.* Modulation on electrostatic potential of passivator for highly efficient and stable perovskite solar cells. *Adv. Funct. Mater.* **33**, 2213123 (2023).
6. Yang T*, et al.* One-stone-for-two-birds strategy to attain beyond 25% perovskite solar cells. *Nat. Commun.* **14**, 839 (2023).
7. Cho E*, et al.* Highly improved photocurrent density and efficiency of perovskite solar cells via inclined fluorine sputtering process. *Adv. Funct. Mater.* **33**, 2301033 (2023).
8. Zhao W*, et al.* Laser derived electron transport layers with embedded p–n heterointerfaces enabling planar perovskite solar cells with efficiency over 25%. *Adv. Mater.* **35**, 2300403 (2023).
9. Guo H*, et al.* In situ polymerization of cross-linked perovskite-polymer composites for highly stable and efficient perovskite solar cells. *Adv. Energy Mater.* **14**, 2302743 (2024).
10. Cao Y*, et al.* Interface modification by ammonium sulfamate for high-efficiency and stable perovskite solar cells. *Adv. Energy Mater.* **13**, 2302103 (2023).
11. Li K*, et al.* Au nanocluster assisted microstructural reconstruction for buried interface healing for enhanced perovskite solar cell performance. *Adv. Mater.* **n/a**, 2310651 (2023).
12. Su H*, et al.* Surface energy engineering of buried interface for highly stable perovskite solar cells with efficiency over 25%. *Adv. Mater.* **36**, 2306724 (2024).
13. Wang X*, et al.* Oriented molecular bridge constructs homogeneous buried interface for perovskite solar cells with efficiency over 25.3%. Adv Mater. **n/a**, e2310710 (2024).
