# Supplementary material for: Inhibiting perovskite decomposition by a creeper-inspired strategy enables efficient and stable perovskite solar cells
Source: Nat Commun. 2024 Jun 18;15:5223. doi: 10.1038/s41467-024-49617-y (PMC11189488; doi:10.1038/s41467-024-49617-y)
Supplement: Supplementary file 3 — Solar Cells Reporting Summary [file 41467_2024_49617_MOESM3_ESM.pdf]

nature portfolio

Corresponding author(s): Meicheng Li

Solar Cells Reporting Summary

Nature Portfolio wishes to improve the reproducibility of the work that we publish. This form is intended for publication with all accepted papers reporting the characterization of photovoltaic devices and provides structure for consistency and transparency in reporting. Some list items might not apply to an individual manuscript, but all fields must be completed for clarity.

For further information on Nature Research policies, including our data availability policy, see Authors & Referees.

Experimental design

Please check the following details are reported in the manuscript, and provide a brief description or explanation where applicable.

1. Dimensions

Area of the tested solar cells

☒ Yes — The aperture area is 0.08 cm2 , which can be found in main text.
☐ No — *Explain why this information is not reported/not relevant.*

Method used to determine the device area

☒ Yes — The area is determined by a mask with an opening area of 0.08 cm2
☐ No — *Explain why this information is not reported/not relevant.*

2. Current-voltage characterization

Current density-voltage (J-V) plots in both forward and backward direction

☒ Yes — We provide J-V plots in both forward ward and backward direction in the main text Fig 4.
☐ No

Voltage scan conditions

☒ Yes — The voltage scan range is from 1.2 V to -0.1 V, the scan speed of 0.06 V/s and dwell time of 5 ms.
☐ No — *Explain why this information is not reported/not relevant.*

Test environment

☒ Yes — The current-voltage characterization was performed in air at room temperature.
☐ No — *Explain why this information is not reported/not relevant.*

Protocol for preconditioning of the device before its characterization

☐ Yes — *Provide a description of the protocol.*
☒ No — No preconditioning is required before characterization.

Stability of the J-V characteristic

☒ Yes — The evolution of photocurrent with time at maximum power point is provided in Fig.4i.
☐ No — *Explain why this information is not reported/not relevant.*

3. Hysteresis or any other unusual behaviour

Description of the unusual behaviour observed during the characterization

☒ Yes — No unusual behaviour was observed.
☐ No — *Explain why this information is not reported/not relevant.*

Related experimental data

☒ Yes — Related experimental data is shown in Fig. 4e, h, and Fig. 5c.
☐ No — *Explain why this information is not reported/not relevant.*

4. Efficiency

External quantum efficiency (EQE) or incident photons to current efficiency (IPCE)

☒ Yes — External quantum efficiency is provided in supplementary Fig 12.
☐ No — *Explain why this information is not reported/not relevant.*

A comparison between the integrated response under the standard reference spectrum and the response measure under the simulator

☒ Yes — The integrated current density matches with the J-V curves.
☐ No — *Explain why this information is not reported/not relevant.*

April 2023

For tandem solar cells, the bias illumination and bias voltage used for each subcell

☐ Yes
☒ No

Provide a description of the measurement conditions.

No tandem solar cell is covered in this work.

5. Calibration

Light source and reference cell or sensor used for the characterization

☒ Yes
☐ No

The current-voltage characterization were performed under simulated AM1.5G illumination (100 mW/cm2) using a 150 W Class AAA solar simulator (XES-40S1, SAN-EI). The light intensity was calibrated by the National Institute of Metrology certified silicon solar cell.

Explain why this information is not reported/not relevant.

Confirmation that the reference cell was calibrated and certified

☒ Yes
☐ No

The standard silicon solar cell was calibrated by the National Institute of Metrology, China.

Explain why this information is not reported/not relevant.

Calculation of spectral mismatch between the reference cell and the devices under test

☒ Yes
☐ No

The standard silicon solar cell calibrated by the National Institute of Metrology, China was used to calibrate the light intensity.

Explain why this information is not reported/not relevant.

6. Mask/aperture

Size of the mask/aperture used during testing

☒ Yes
☐ No

The aperture area of mask is 0.08 cm2.

Explain why this information is not reported/not relevant.

Variation of the measured short-circuit current density with the mask/aperture area

☒ Yes
☐ No

All J-V curves were measured with aperture area of 0.08 cm2.

Explain why this information is not reported/not relevant.

7. Performance certification

Identity of the independent certification laboratory that confirmed the photovoltaic performance

☒ Yes
☐ No

The certificated PCE was obtained in the National Institute of Metrology, China (NIM, China), and the corresponding result is provided in the supplementary Fig. 13.

Explain why this information is not reported/not relevant.

A copy of any certificate(s)

☐ Yes
☐ No

The certificated result is provided in Supplementary Fig. 13.

Explain why this information is not reported/not relevant.

8. Statistics

Number of solar cells tested

☒ Yes
☐ No

Histograms of the PCE value among 40 PSCs were shown in Fig.4d, g and Supplementary Fig. 11.

Explain why this information is not reported/not relevant.

Statistical analysis of the device performance

☒ Yes
☐ No

The statistical analysis is shown in Fig. 4d, g and Supplementary Fig. 11.

Explain why this information is not reported/not relevant.

9. Long-term stability analysis

Type of analysis, bias conditions and environmental conditions

☒ Yes
☐ No

SEM and XRD were used to characterize the long-term stability of films. The unencapsulated devices used to measure long-term stability were stored under ambient conditions of ~10% RH and 20-30°C. Operational stability measurement of the unencapsulated devices were performed under continuous 1-sun illumination in N2 glove box. The unencapsulated devices were stored under ~85°C and ~25% RH to measure long-term thermal stability. The stability of encapsulated devices stored in damp environment (~85% RH and ~23°C) were measured. The correlation analysis are presented in Fig. 6, and supplementary Fig. 18-19.

Explain why this information is not reported/not relevant.
